# ShRNA-mediated gene silencing of MTA1 influenced on protein expression of ER alpha, MMP-9, CyclinD1 and invasiveness, proliferation in breast cancer cell lines MDA-MB-231 and MCF-7 in vitro

**DOI:** 10.1186/1756-9966-30-60

**Published:** 2011-05-19

**Authors:** Qingming Jiang, Hui Zhang, Ping Zhang

**Affiliations:** 1 Department of Pathology, School of Basic Medicine Sciences, Chong Qing University of Medical Sciences, Chong Qing, 400016, China

## Abstract

**Background:**

MTA1(metastasis associated-1) is a tumor metastasis associated candidate gene and overexpression in many human tumors, including breast cancer. In this study, we investigated depressive effect on MTA1 by MTA1-specific short hairpin RNA(shRNA) expression plasmids in human breast cancer cell lines MDA-MB-231 and MCF-7, and effect on protein levels of ER alpha, MMP-9, cyclinD1, and tumor cell invasion, proliferation.

**Methods:**

ShRNA expression vectors targeting MTA1 was constructed and transfected into human breast cancer cell lines MDA-MB-231 and MCF-7. The transfection efficiency was evaluated by fluorescence microscopy, mRNA levels of MTA1 were detected by reverse transcription-polymerase chain reaction (RT-PCR), protein levels of ER alpha, MMP-9 and cyclinD1 were detected by Western blotting, respectively. Tumor cells invasive ability were evaluated by Boyden chamber assay, the cells proliferation were evaluated using cell growth curve and MTT analysis, the cell cycle analysis was performed using flow cytometry.

**Results:**

Down-regulation of MTA1 by RNAi approach led to re-expression of ER alpha in ER-negative breast cancer cell lines MDA-MB-231, and reduced protein levels of MMP-9 and CyclinD1, as well as decreased tumor cell invasion and proliferation, more cells were blocked in G0/G1 stage(P < 0.05). However, after inhibiting mRNA levels of MTA1, protein expression of ER alpha, MMP-9, cyclinD1 and the changes of cancer cells invasiveness, proliferation, cells cycle were no statistical difference in ER-positive human breast cancer cell lines MCF-7 (P > 0.05).

**Conclusions:**

ShRNA targeted against MTA1 could specifically mediate the MTA1 gene silencing and consequentially recover the protein expression of ER alpha, resulting in increase sensitivity of antiestrogens, as well as suppress the protein levels of MMP-9 and cyclinD1 in ER-negative human breast cancer cell lines MDA-MB-231. Silencing effect of MTA1 could efficiently inhibit the invasion and proliferation in MDA-MB-231 cells. The shRNA interference targeted against MTA1 may have potential therapeutic utility in human breast cancer.

## Background

Breast cancer is one of the most commonly seen, malignant tumors in human, and the incidence rate is gradually increasing year by year. Based on the GLOBOCAN 2008 estimates, breast cancer is the most frequently diagnosed cancer and the leading cause of cancer death among females, accounting for 23% of the total cancer cases and 14% of the cancer deaths[1]. Currently, combined therapy, which primarily focused on surgical removal, chemotherapy and endocrine therapy based on tamoxifen, is employed for most cases of breast cancer. The poor prognosis of the patients with advanced stage breast cancer is due mainly to the progression and metastasis of the disease after the standard surgical treatment. Clearly, a better understanding of the molecular mechanisms underlying the progression of breast cancer is needed to control the disease. With the development of molecular biology and genetic engineering, the gene therapy is the research focus on prevention and treatment of tumor. Currently, gene therapies for tumor include gene replacement, antisense nucleic acid technique, cytokine gene therapy, and RNA interference technique mostly focused in recent years. RNA interference is the most effective gene silencing technique, while being simple, effective, and specific as its advantages. The short hairpin RNA (shRNA) could automatically be processed to become small interfering RNA(siRNA) to silence target gene, and it was proven to be more stable than siRNA[2].

Metastasis associated antigen 1 (MTA1) is a tumor metastasis associated candidate gene, it was originally identified by differential screening of a cDNA library from highly metastatic and non-metastatic rat mammary adenocarcinoma cell lines[3,4]. Overexpression of MTA1 plays an important role in tumorigenesis and tumor aggressiveness, especially tumor invasiveness and metastasis, including breast cancer[5]. The ER expression status is related to a variety of histologic characteristics of breast cancer. Most tumors with low grades are ER-positive but, in contrast, tumor demonstrating histologic evidence of poor tumor differentiation are frequently ER-negative[6]. Molecular characterizations and epidemiological studies for breast cancer showed that it was important roles of ER in tumorigeness and progression. ER subtypes, ER alpha(ER_α_), was known to mediate estrogen signaling; and the function as ligand-dependent transcription factors. At the molecular level, the consequence of ER activation appears to be alterations in transcriptional activity and expression profiles of target genes. A number of genes, including cyclinD1, are regulated by ER alpha[7].

In this study, two shRNA plasmid vectors against MTA1, which could persistently generate siRNA inside cells, were constructed and transfected into the breast cancer cell lines MDA-MB-231 and MCF-7. Its effect on protein expression of estrogen recepter alpha(ER_α_), matrix metalloproteinase 9(MMP-9), cyclinD1, and on cancer cells invasion, proliferation and cell cycle cell in two cell lines were investigated.

## Methods

### Cell lines and culture

The human breast cancer cell lines MDA-MB-231 and MCF-7 were kindly supplied by professor Wei-xue Tang(Department of Pathology Physiology, School of Basic Medicine Sciences, Chong Qing University of Medical Sciences, China). All cells were cultured in RPMI 1640 medium (Gibio BRL, USA) supplemented with 10% fetal bovine serum,100 U/ml penicillin, and 100 μg/ml streptomycin. The cells were plated in a fully humidified atmosphere containing 5% CO2/95% air at 37°C. The cells in exponential phase of growth were experimentized after digestion with 0.1% pancreatic enzyme.

### Construction of shRNA expression vector for MTA1

According to principle of shRNA, enzyme inciding site of vector pGenesil-1 and exon of MTA1 (GeneBank, No. NM004689) in GeneBank, two target DNA fragments were designed and constructed to coding region 194~216 bp and 529~551 bp for MTA1. The first pair sense:5'-GCAACCCTGTCAGTCTGCTATAA-3', and anti-sense: 5'-TTATA GCAGACTGACAGGGTTGC-3', the second pair: sense:5'-GGCAGACATCACCGA CTTGTTAA-3', and antisense:5'-TTAACAAGTCGGTGATGTCTGCC-3', loop-stem structure was nonhomologous base (TCTCTTGAA), it was non-complementary to MTA1.enzyme inciding sites of BamHI and HindIII were constructed into extreme of oligonucleotides fragment, specificity of constructed oligonucleotides fragments were analyzed by BLAST. The sequence as follow, the first pair:sense:5'-AGCTTAAAAAG CAACCCTGTCAGTCTGCTATAA***TTCAAGAGA***TTATAGCAGACTGACAGGGTT GCGG-3', antisense: 5'-GATCCCGCAACCCTGTCAGTCTGCTATAA***TCTCTTGA A***TTATAGCAGACTGACAGGGTTGCTTTTTA-3', the second pair:sense:5'-AGCTT AAAAAGGCAGACATCACCGACTTGTTAA***TTCAAGAGA***TTAACAAGTCGGT GATGTCTGCCGG-3', and antisense: 5'-GATCCCGGCAGACATCACCGACTTGT TAA***TCTCTTGAA***TTAACAAGTCGGTGATGTCTGCCTTTTTA-3'(italic word is loop). Sense and antisense oligonucleotides were annealed, pGenesil-1 vector was cut off by BamHI and HindIII, then products were recovered and purified. shRNA oligonucleotides fragment and pGenesil-1 vector were ligated(mole ratio:3:1), recombinant plasmid was named for pGenesil-1/MTA1-shRNA(pGM). Then, the recombinant plasmid were transformed into competence bacillus coli, and bacterium were cultured, recombinant plasmid were extracted, purified and cut off using restrictive enzyme BamHI, HindIII and XbaI for identification. Then recombinant plasmid concentration were measured, purified and stored in -20°C refrigerator. Some of the constructed pGenesil-1/MTA1 shRNA expression plasmid were sent to Shang Hai Ding An Corp in China for sequencing.

### Transfection with shRNA/MTA1 expression vector

Two breast cancer cells were divided into four groups: the first group was blank control(no transfection), the second group was negative control(transfection with empty vector pGenesil-1, pG), the third group was pGM1(transfection with the first pGenesil-1/MTA1-shRNA), the forth group was pGM2(transfection with the second pGenesil-1/MTA1-shRNA). MDA-MB-231 and MCF-7 cells were plated in six-well plates at a density of 3 × 10^5 ^cells per well and incubated overnight. Cells were transfected with pG, pGM1, pGM2 and blank control, using Lipofectamine 2000 (Invitrogen, Carlsbad, CA, USA) according to the manufacturer's instructions, respectively. GFP was observed and taken photos by fluorescence microscope at transfection 36 hours. Forty-eight hours after transfection, MDA-MB-231 and MCF-7 cells were diluted to 1:10 for passage and neomycin resistance clones were selected in the medium containing 500 μg/ml G418(Gibco BRL, Grand Island, NY, USA) for one week. Then, the density of G418 changed to 250 μg/ml. The positive clones were picked up and expanded to establish cell lines after maintaining to select for 2 weeks. The stable transfection cell clones were verified for RT-PCR and Western blot analysis.

### Selection of recombinant plasmid by RT-PCR

Total RNA was extracted using Trizol reagent (Gibco BRL, USA) and quantified using UV absorbance spectroscopy on 1% agarose-formaldehyde gels. The reverse transcription reaction was performed using 2 μg total RNA with M-MLV reverse transcriptase, the newly synthetized cDNA template (2 μl) was amplified by PCR for MTA1(GeneBank NO. NM004689), the forward and reverse primers were 5'-AGCTA CGAGCAGCACAACGGGGT-3'(forward), 5'-CACGCTTGGTTTCCGAGGAT-3' (reverse), the amplified products for PCR were 290 bp. The PCR cycling program was 94°C for 5 minutes, then 35 cycles at 94°C for 30 seconds, 58.5°C for 45 seconds, 72°C for 90 seconds, and a final extension at 72°C for 10 min. The control was 18SrRNA(GeneBank, NO. X67238), the forward and reverse primers were 5'-TTGAC GGAAGGGCACCACCAG-3', reverse: 5'-GCACCACCAACGGAATCG-3', the amplified products were 130 bp. The PCR cycling program was 94° for 5 minutes, 25 cycles at 94°C for 5 seconds, 56.5°C for 5 seconds, 72°C for 20 seconds, and a final extension at 72°C for 10 min. the PCR products were electropheresed on 1.5% agarose gels and PCR fragments were visualized by UV illumination (Gel Doc 1000, BIO RAD corp, USA) stained with ethidium bromide. The fluorescence intensity of 18SrRNA fragments served as the criterion for MTA1, To intercomparing two recombinant plasmid constructed, one of the better inhibitory efficiency was done next experiments.

### Western blot analysis for ER alpha, MMP-9 and CyclinD1

After extraction from the culture medium, cells were washed three times with PBS, cells per 10 mg were lysed in 100 μl of cells lysis buffer(mammalian protein extraction reagent, Pierce, 78503, USA) for 10 minutes, then centrifugated at 15300 rpm for 15 minutes at 4°C, got the supernatant to measure protein concentration. Protein per 60 μg were done electrophoresis experiment in 10% SDS-PAGE at 4°C, steady flow(10 mA in composition gel, 15 mA in separation gel), then transfered into nitrocellulose membranes in ice bath at voltage-sdtabilizing (Gibco BRL, USA). The membranes were blocked with 5% skim milk in TBST (20 mmol/L Tris-Hcl at PH 8.0, 150 mmol/L NaCl, and 0.05% Tween 20) for 1 hour at room temperature, the membranes were probed with 1:500 dilution of anti-ER alpha antibodies (Sc-542, Santa Cruz, USA), 1:400 mouse monoclonal antibody to MMP-9 (Sc-21733, Santa Cruz, USA) and 1:500 mouse monoclonal antibody to cyclinD1 (Sc-8396, Santa Cruz, USA) at 4°C overnight, followed by incubation in a 1:2000 dilution of secondary antibodies conjugated to horseradish peroxidase (Zhongshan Golden Bridge Biotechnology, China). Protein bands were detected using ECL detection system (Zhongshan Golden Bridge Biotechnology, China), and β-actin staining served as the internal standard for the membranes. All of the Western blots were performed at least three times.

### Boyden Chamber Assays

Cells groups described previously, Boyden chambers(containing transwell filter membrane, Corning Costar Corp, Cambridge, MA) invasion assay was carried out as instruction, as described previously with a slight modification, suspensions of 1 × 10^5 ^cells in 200 μl of RPMI1640 containing 0.1% fetal calf serum were plated on the upper compartment of the chamber. Conditioned medium(800 μl, supernatant fluid that cultured NIH3T3 cells with serum-free medium) was placed in the lower compartment. After 24 h at 37°C, noninvasive cells on the upper surface of the filters were removed completely with a cotton swab carefully. The filters were then fixed with 95% alcohol for 15 minutes and stained with 4% trypan blue. Cells on the lower surface were photographed under a microscope, and counted. The data were expressed as mean ± S.D. invasion index: cells through Matrigel/cells without Matrigel ×100%. Experiment in every filter was performed at least three times.

### Cells proliferation state analysis

Cell groups described previously, 24 filters were seed with 5 × 10^3 ^cells per filter, cells in three filters were digest by trypsin per 24 hours and counted cells number, measured mean value. continued to observe for 7 days, drew growth curve. The 96 filter were seed with 2 × 10^3 ^cells/filter, and cells were cultured for 24, 48, 72 and 96 hours, respectively, then added 20 ul MTT to cells and cultured for 4 hours. After removing the culture medium and adding 200 ul DMSO to cells, cells were shaken well for 10 minutes, and the absorbance(A_570 nm_) were detected by enzyme linked immunodetection analysator. Cells growth curve were drawn after collection datas of A_570 nm _at 4 time points successfully. The zero setting was the blank control added culture medium, every experiment was repeated three times.

### Cell cycle analysis by Flow Cytometry

A total of 1 × 10^6 ^cells at logarithmic phase were seeded into a 6-well culture plate. Then cells were harvested by centrifugation and washed twice with ice-cold PBS (pH 7.4). The cells were fixed in ice-cold 70% ethanol at least for 24 h at 4°C. Next, the cells were washed twice with PBS and resuspended in lml DNA staining solution (50 μg/ml propidium iodide(PI) and 100 μg/ml RNase A in PBS)for 30 min. Analysis of cell cycle distribution was performed by Flow Cytometer and analyzed by Cell Quest software package. Every experiment was repeated three times.

### Image analysis

The image analysis for RT-PCR and Western blot were performed by Quantity One 4.5 image analytical system, optical density ratio(ODR) of strap indicated as follow: ODR_Mta1_: MTA1/18SrRNA, ODR_E_: ER alpha/β-Actin, ODR_MMP-9_: MMP-9/β-Actin, ODR_C_:CyclinD1/β-Actin.

### Statistical analysis

The statistical significance of differences in mean values was assessed using Student's t test with SPSS 11.0 statistic software. P < 0.05 was considered statistically significant. Average values were expressed as mean ± standard deviation (SD).

## Results

### The construction of pGenesil-1/MTA1 shRNA expression plasmid

**Figure 1:**
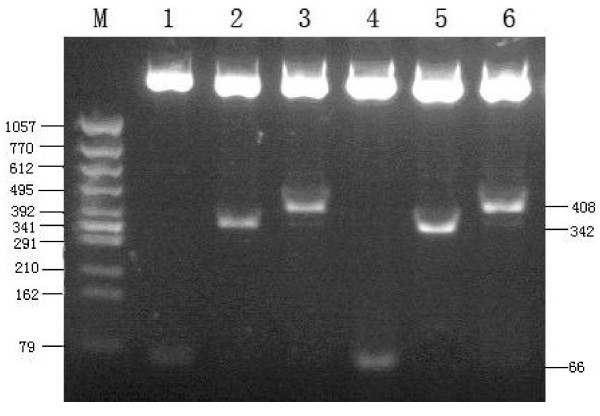
**Restrictive enzyme incision analysis for pGensil-1/MTA1 shRNA plasmid using RT-PCR**. M: DNA Marker. lane 1: pGenesil-1/MTA1 shRNA(pGM1) plasmid was cut off by BamHI and HindIII. lane 2: pGenesil-1/MTA1 shRNA(pGM1) plasmid was cut off by BamHI and XbaI.lane 3: pGenesil-1/MTA1 shRNA(pGM1) plasmid was cut off by HindIII and XbaI. lane 4: pGenesil-1/MTA1 shRNA(pGM2) plasmid was cut off by BamHI and HindIII. lane 5: pGenesil-1/MTA1 shRNA(pGM2) plasmid was cut off by BamHI and XbaI. lane 6: pGenesil-1/MTA1 shRNA(pGM2) plasmid was cut off by HindIII and XbaI.

The recombinant plasmids were cut off by restriction enzyme Xba, BamHⅠand HindⅢ, The band about 66 bp was cut off using BamHⅠand HindⅢ on 0.8% agarose gel electrophoresis, the band about 342 bp was cut off using XbaⅠand BamHⅠ, the band about 408 bp was cut off using XbaⅠand HindⅢ (Figure 1). The results of incision with restriction endonucleases and sequencing showed correct plasmids.

### Observation of transfection results

**Figure 2:**
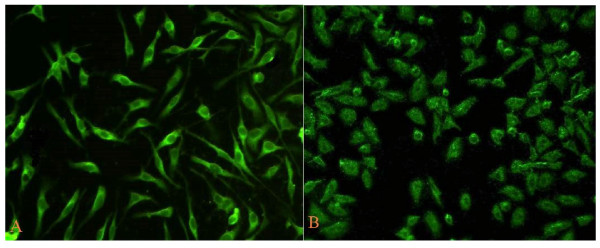
**The expression of GFP in breast cancer cells MDA-MB-231 and MCF-7 transfected with pGenesil-1/MTA1 shRNA recombinant plasmids under fluorescent microscope**. A. MDA-MB-231 cells transfected with pGenesil-1/MTA1 shRNA plasmids for 36 h. B. MCF-7 cells transfected with pGenesil-1/MTA1 shRNA plasmids for 36 h.

After transfection with the recombinant plasmid, the breast cancer cell lines MDA-MB-231 and MCF-7 showed green luminescence(green fluorescent protein, GFP), suggesting the correct expression of pGenesil-1/MTA1 shRNA (Figure 2).

### ShRNA targeting MTA1 inhibited MTA1 mRNA expression in MDA-MB-231 and MCF-7 cells

**Figure 3:**
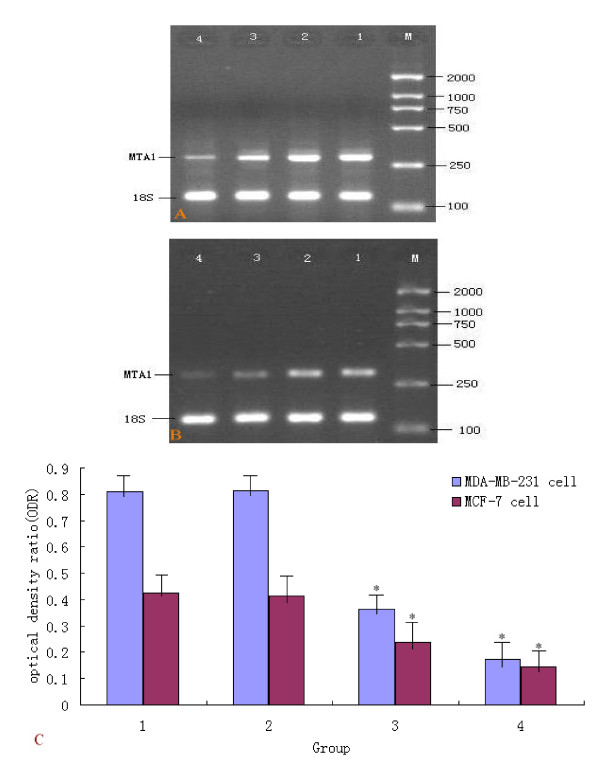
**MTA1 specific shRNAs results in the reduction of MTA1 mRNA levels in MDA-MB-231 and MCF-7 cells**. A: mRNA levels of MTA1 in MDA-MB-231. M:DNA Marker. lane 1:Blank control group. lane 2: PG group(empty vector). lane 3: PGM1 group(the first pair pGenesil-1/MTA1-shRNA). lane 4:PGM2 group(the second pair pGenesil-1/MTA1-shRNA). B: mRNA levels of MTA1 in MCF-7. M:DNA Marker. lane 1:Blank control group. lane 2: PG group(empty vector). lane 3:PGM1 group(the first pair pGenesil-1/MTA1-shRNA). lane 4:PGM2 group(the second pair pGenesil-1/MTA1-shRNA). C: Column diagram analysis for mRNA levels of MTA1, MTA1 specific shRNAs resulted in the reduction of MTA1 mRNA levels in MDA-MB-231 and MCF-7 cells (*P < 0.05).

The mRNA expression intensities of goal genes, inhibited by specific shRNAs in the breast cancer cells MDA-MB-231 and MCF-7, were analyzed by semiquantitive RT-PCR. The mRNA levels were normalized by internal control 18SrRNA. In MDA-MB-231 cells, The mRNA optical density ratio(ODR: MTA1/18SrRNA) of MTA1 in the blank control, negative control and test groups (pGM1, pGM2) were 0.8097 ± 0.0173, 0.8119 ± 0.0367, 0.3623 ± 0.0087 and 0.1742 ± 0.0094, respectively. The statistical analysis showed that MTA1 mRNAs of MDA-MB-231 cells in the pGM1 and pGM2 groups were down-regulated significantly after transfection with either plasmids pGM1 or pGM2, compared with that in the blank group(*P *< 0.05). The inhibition rates were 55.3% and 78.5% in the pGM1 and pGM2 group, respectively. In MCF-7 cells, ODR in pGM1 and pGM2 group were 0.2386 ± 0.0018 and 0.1455 ± 0.0075, respectively. Compared to blank control group (ODR:0.4236 ± 0.0069) and negative control(ODR:0.4148 ± 0.0058), there were statistical difference(P < 0.05). MTA1 mRNA inhibition rate for pGM1 and pGM2 were 43.7%, 65.7%. Thus, MDA-MB-231/pGM2 and MCF-7/pGM2 cell clones were chosen for further experiments. (Figure 3)

### Influence of pGenesil-1/MTA1 shRNA vectors on ER alpha, MMP-9 and CyclinD1 protein expression in MDA-MB-231 and MCF-7 cells by Western blot analysis

**Figure 4:**
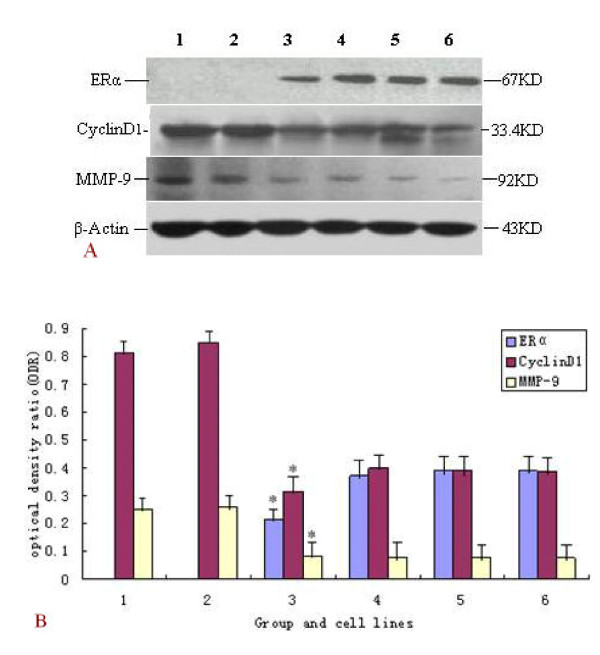
**Western blot analysis for ER alpha, CyclinD1 and MMP-9 in MDA-MB-231 and MCF-7 cells**. A: Western blot analysis for ER alpha, CyclinD1 and MMP-9. lane 1: blank control group in MDA-MB-231 cells. lane 2: PG group (empty vector) in MDA-MB-231 cells. lane 3:PGM2 group (the second pair pGenesil-1/MTA1 shRNA plasmid) in MDA-MB-231 cells. lane 4: blank control group in MCF-7 cells. lane 5: PG group(empty vector) in MCF-7 cells. lane 6:PGM2 group in MCF-7 cells. B: Column diagram analysis for protein expression of ER alpha, cyclinD1, MMP-9 in MDA-MB-231 and MCF-7 cells by Western blotting.1-3: blank control group, PG group and PGM2 group in MDA-MB-231 cells, respectively. 4-6: blank control group, PG group and PGM2 group in MCF-7 cells respectively. As shown in the Figure, ER alpha protein expression was recovered positive in ER_α_-negative breast cancer cell lines MDA-MB-231, MMP-9 and CyclinD1 protein levels were down-regulated(*P < 0.05). But in ER_α_-positive breast cancer cells MCF-7, protein levels of ER alpha, MMP-9 and CyclinD1 had no distinct difference in three groups (P > 0.05).

Results in two breast cancer cells by Western blot ananlysis indicated that, ER alpha was recovered positive in ER-negative human breast cancer cell lines MDA-MB-231, and protein levels of MMP-9 and CyclinD1 were down-regulation (P < 0.05). However, in ER alpha-positive breast cancer cells MCF-7, protein expression levels of ER alpha, MMP-9 and CyclinD1 had no distinct difference in three groups(P > 0.05). (Figure 4)

### MTA1 silencing reduces the invasive ability of MDA-MB-231 cells in vitro

**Figure 5:**
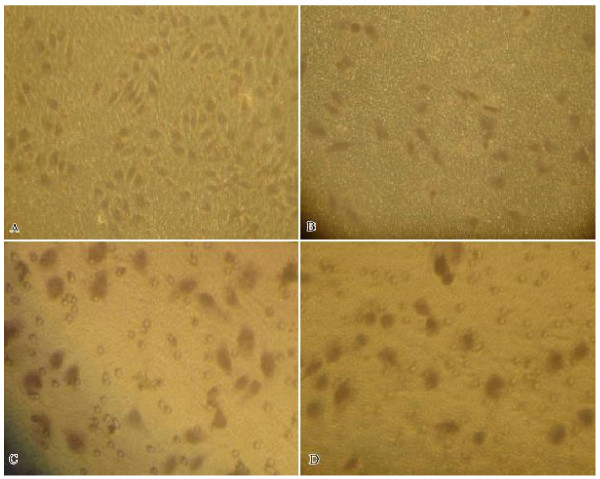
**Effects of MTA1 specific shRNA on invasion in MDA-MB-231 and MCF-7 cells**. A: MDA-MB-231 cells passed through the filter and attached to the lower side of the filter (400×)before silencing MTA1. B: MDA-MB-231 cells passed through the filter and attached to the lower side of the filter (400×) after silencing MTA1 C: MCF-7 cells passed through the filter and attached to the lower side of the filter (400×) before silencing MTA1. D: MCF-7 cells passed through the filter and attached to the lower side of the filter (400×) after silencing MTA1.

The effects of inhibiting MTA1 gene on invasion of breast cancer cells were evaluated by Boyden chamber migration assay. The invasion index before silencing MTA1 in MDA-MB-231 and MCF-7 cells were 76.3 ± 2.4%, 25.6 ± 1.9%, respectively, the difference was obvious(P < 0.05). After silencing MTA1 gene in MDA-MB-231 cells, the invasion index was 27.2 ± 2.1%, compared to before transfection, the statistics difference was obvious(P < 0.05). But in MCF-7 cells, invasion index was 23.3 ± 1.6% after silencing MTA1, compared to blank control, it's no statistics difference(P > 0.05). The invasion index in MDA-MB-231 and MCF-7 cells treated with empty vector were 73.2 ± 2.0%, 23.1 ± 2.1%, compared to blank control, its' no statistics difference(P > 0.05), respectively. (Figure 5)

### MTA1 silencing reduced the proliferation in MDA-MB-231 cells in vitro

**Figure 6:**
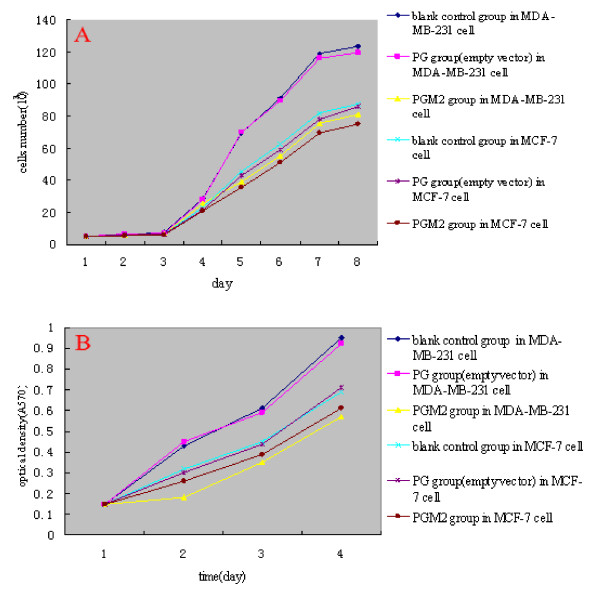
**Cells growth curve and MTT analysis for MDA-MB-231 and MCF-7 cells**. A: cells growth curve analysis for MDA-MB-231 and MCF-7 cells. B: MTT analysis for MDA-MB-231 and MCF-7 cell. compared to blank control group and PG group(empty vector), the cells growth velocity and proliferation speed descend obviously after silencing MTA1 gene(P < 0.05). But in MCF-7, after silencing MTA1 gene, it's no obvious diference in cells growth velocity and proliferation speed(P > 0.05).

Next, we analyzed the growth velocity and proliferation of blank control group, PG group and PGM2 group. Compared with blank control group, after silencing MTA1 in MDA-MB-231 cells, the growth velocity and proliferation speed of cells reduced obviously(P < 0.05). But in MCF-7 cells, it's no statistical difference in growth velocity and proliferation speed of cells after silencing MTA1(P > 0.05). The results in negative group showed no effects on two breast cancer cells(Figure 6).

### Influence of silencing MTA1 mRNA expression on cell cycle

**Figure 7:**
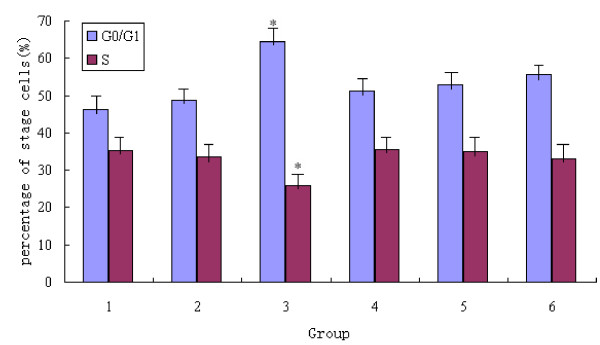
**Column diagram analysis for effect of inhibition MTA1 gene on cell cycle**. 1-3: blank control group, PG group(empty vector), PGM2 group in MDA-MB-231 cells; 4-6: blank control group, PG group(empty vector), PGM2 group in MCF-7 cells. The results indicated that more MDA-MB-231 cells were blocked in G0/G1 stage after inhibition MTA1 gene by pGenesil-1/MTA1 shRNA plasmid(*P < 0.05), but in MCF-7 cells, there was no statistically significant difference of effect on cell cycle(P > 0.05).

After silencing MTA1 mRNA expression in MDA-MB-231 and MCF-7 cells, cell cycle was examined. The mean value of the experiments was shown in Figure 7. In MDA-MB-231 cells, the percentage of G0/G1 stage cells in PGM2 group was 64.45 ± 1.39%, compared to blank control group and PG group(46.40 ± 1.88%, 48.90 ± 1.54%), the statistical difference was significant(P < 0.05). The percentage of S stage cells in PGM2 group was 25.99 ± 0.62%, compared to blank control group and negative group(35.14 ± 1.52%, 33.67 ± 1.32%), the statistical difference was significant, (P < 0.05). But in MCF-7 cells, the percentage of G0/G1 stage cells in blank control group, negative control group and PGM2 group were 51.25 ± 2.07%, 52.83 ± 1.76%, 55.75 ± 1.69%, and the percentage of S stage cells in blank control group, PG group and PGM2 group were 35.43 ± 1.52%, 34.88 ± 2.12%, 32.95 ± 2.29%, there were no statistically significant difference(P > 0.05). The results indicated that, more MDA-MB-231 cells were blocked in G0/G1 stage after inhibiting MTA1 gene by pGenesil-1/MTA1 shRNA.

## Discussion

Breast cancer has the characteristics of powerful invasion ability and early metastatic property, which are the primary reasons for failure in therapy. To research the molecular mechanisms for invasion and metastasis of breast cancer cells, as well as finding treatment target site, has significant meaning for improvement the prognostic outcome. Currently, researches that involved the gene such as MTA1, which were related to tumor metastasis, revealed that the expression level was closely related to the metastatic ability.

MTA1 is a tumor metastasis associated candidate gene. It was cloned and selected from the 13762NF rat mammary adenocarcinoma cell lines with different spontaneous metastatic potentials by Toh et al in 1994[4]. the cDNA length of MTA1 was about 2.8 kb, encoded 703 amino acids and phosphoprotein of 80 kD. In 2000, Nawa et al[8] detected mta1 correlated series MTA1 in two breast cancer metastasis system, meanwhile, and found that MTA1 gene located on 14q32 of chromosome by antisense phosphorothioate oligonucleotides. Zhu X et al[9] found that overexpression of MTA1 was associated with tumor progression and clinical outcome in patients with NSCLC. MTA1 overexpression was detected in node-negative esophageal cancer and was significantly correlated with shorter disease-free interval[10]. It's indicated that MTA1 gene involved in the critical molecule mechanism of tumor infiltration and metastasis.

RNA interference(RNAi) is a ubiquitous mechanism of eukaryotic gene regulation and an excellent strategy for specific gene silencing. The specificity of RNAi is determined by 21-23 nt RNA duplexes, referred to as micro-RNA (miRNA) or small interfering RNAs (siRNA). ShRNA is formed by hairpin structures and stretches of double-stranded RNA, which will be cleaved by the ribonuclease dicer to produce mature miRNA inside the targeted cells. After unwinding, one of the strands becomes incorporated into the RNA-induced silencing complex (RISC) and guides the destruction or repression of complementary mRNA. Recently the vector-based approach of shRNA interference has been developed in order to achieve stable, long-term, and highly specific suppression of gene expression in mammalian cells. These shRNA expression vectors have many advantages: they can be stably introduced into cells and persistently effective, either as selectable plasmids or as retroviruses. They are relatively cheap to generate. These vectors are often under the control of an RNA polymerase III promoter such as U6 or H1. They can transcribe and generate siRNA continuously and the gene silencing effect can last persistently inside the cells. These findings have opened a broad new avenue for the analysis of gene function and gene therapy[2,11]. Here, we successfully transfected two shRNAs targeting MTA1 gene into human breast cancer cell lines MDA-MB-231 and MCF-7. Two stable cell clones pGM1 and pGM2 were obtained. MTA1 expression was effectively inhibited at mRNA levels by pGM1 and pGM2, while the pGM1 was less efficient. These results indicated that shRNA targeting different sites of the same mRNA might be different in silencing efficiency.

Homo sapien estrogen receptor alpha(ER alpha) was first cloned by Green et al[12] in 1986. Estrogen has crutial roles in the proliferation of cancer cells in reproductive organs such as breast and uterus, The estrogen-stimulated growth in tumor cells as well as in normal cells requires estrogen receptor(ER). The ER expression status is in variety of histologic characteristics of breast cancer. Most tumor with low grades are ER-positive but, in contrast, tumors demonstrating histologic evidence of poor tumor differentiation are frequently ER-negative. Breast tumors which lack any ER expression often reveal more aggressive phenotypes[5]. In our experiments, after silencing MTA1 gene by expression vector pGenesil-1/MTA1 shRNA, ER alpha was detecteded again in ER-negative human breast caner cell lines MDA-MB-231 using Western blot analysis, in contrast, silencing MTA1 gene was no effect on protein expression of ER in ER-positive cell lines MCF-7.

How to regulate expression of ER alpha by MTA1? Most literature indicated that it was regulated on transcription level, especially on chromatin level. Two mechanism as follows: one was chromatin remolding in dependence of ATP, the other was covalent modification in nucleosome. The major study of covalent modification focused on acetylation and deacetylation in N-terminal of histone. N-terminal acetyl could be neutralize by positive ion of histone, and degrade DNA combined to acetylation domain, then open the chromatin structure and promote transcription, on the contrary, deacetylation of histone made chromatin structure become compacting, and restrain transcription. Acetyl was linked to N-terminal of histone by histone acetylase (HAT) catalyzing, then the histone acetyl in N-terminal was hydrolyzed by histone deacetylases(HDACs)[13]. MTA1 was considered one of the nucleosome remodeling and histone deacetylase subunit that possessed nucleosome remodeling and histone deacetylase activity[14]. MTA1 integrated with HDACs tightly and correlated to histone deacetylase, So it was considered aid actuating factor of HDACs to restrain transcription. Talukger et al[15] studied, the molecule mechanism of MTA1 restraining ER alpha expression in breast cancer cells was that MTA1 interacted with MTA1, a cyclin-dependent kinase-activating kinase complex ring finger factor, and regulated estrogen receptor transactivation.

Mazumdar et al[16] studied that, MTA1 restrained CAK-induced ER alpha transcription by histone deacetylase in breast cancer cells, the cells deprived reaction to estrogen and possessed malignant phenotype. The protein expression of ER alpha which was inhibitory state recovered again due to silencing MTA1, the mechanism was correlated to deacetylating of MTA1, so ER alpha resumed to transcription. Sharma et al[17] studied, release of methyl CpG binding proteins and histone deacetylase 1 from the Estrogen receptor alpha promoter could take effect on reactivation in ER alpha-negative human breast cancer cells. The results of our works were in accordance with findings in literature above mentioned.

Previous studies and researches indicated that more direct evidence was obtained with estrogen receptor (ER)-positive breast cancer cell lines in which estrogens were found to stimulate the expression of specific genes and the proliferation of these cells. However, ER-positive tumor cells are poorly metastatic when compared with some ER-negative breast cancer cells. In patients, ER-positive tumors are more differentiated and have lower metastatic potential than ER-negative tumors, suggesting a protective role of the estrogen receptor in tumor progression, and human breast cancer cells are more responsive to antiestrogens[18].

The ability of tumor cells to invade surrouding tissue is one of the most important features of the malignant phenotype[19]. Degradation of the basement menbrane invasion of underlying connective tissue have long been the histologic criteria for diagnosis of carcinoma. Invading tumor cells must secrete proteolytic enzymes to degrade basement membranes. Matrix metallopproteinases(MMPs) are a family proteolytic enzymes that degrade specific basement menbrane components. One member of this family, MMP-9 was up-regulation in invasive cancers, including breast cancer. After silencing of MTA1 gene, we investigated the alteration of tumor cells invasiveness using Boyden chamber assay mentioned in Albini's[20] literature. The results showed that tumor cells invasiveness was suppressed in ER-negative cells MDA-MB-231. At the same time, the protein expression of MMP-9 was analyzed using western blotting. The results showed that protein expression of MMP-9 was down-regulated in MDA-MB-231 cells transfected with expression vector pGenesil-1/MTA1 shRNA. However, the tumor cells invasiveness and protein levels of MMP-9 were no statistical difference in ER-positive cells MCF-7. David L et al[21] studied that c-fos/ER fusion protein activation produced MMP-9 down-regulation and concomitant reduction in tumor cell invasion. The reduction in MMP-9 activity was mediated at the transcriptional level by the proximal AP-1 site of the promoter. Vinodhkumar et al[22] found that, depsipeptide a histone deacetylase inhibitor could down-regulate levels of matrix metalloproteinases 9 mRNA and protein expressions in lung cancer cells (A549). MTA1, a aid activation factor of histone deacetylase might down-regulate MMP-9 expression level by direct manner and by a c-fos/ER fusion protein indirectly.

In carcinogenesis, one of the important steps is to obtain proliferative capacity without external stimuli, usually as a consequence of oncogene activation; cyclinD1 and ER are well-known for their involvement in the cell proliferative activity. CyclinD1, known as a key cell cycle regulator, regulates the transition of G1 and S phase. Silence of MTA1 might inhibit expression of cyclinD1. The results indicated that, after stable transfection with recombinant plasmid in ER-negative cells MDA-MB-231, mRNA expression of MTA1 was down-regulated, this result led to that cell growth curve shifted right, cell population double time prolongated, and cells growth rate degraded, obviously. However, the same results didn't appear in blank control group and negative group. The results indicated that, the silence of MTA1 might reduce cell proliferation ability. Rozita Bagheri-Yarmand's study found that, MTA1 dysregulation in mammary gland epithelium triggered downregulation of the progesterone receptor-B isoform and upregulation of the progesterone receptor-A isoform, resulting in an imbalance in the native ratio of progesterone receptor A and B isoforms. MTA1 transgene also increased the expression of progesterone receptor-A target genes cyclinD1[23].

## Conclusions

In conclusion, our experiments showed that the shRNA targeted against MTA1 could specifically mediate the MTA1 gene silence and consequentially recover the protein expression of ER alpha, resulting in increase sensitivity of antiestrogens, as well as suppress the protein expression of MMP-9 and cyclinD1 in ER-negative human breast cancer cell lines MDA-MB-231. The silence effect of MTA1 could efficiently inhibit the invasion and proliferation of MDA-MB-231 cells. The shRNA interference targeted against MTA1 may have potential therapeutic utility in human breast cancer.

## Competing interests

The authors declare that they have no competing interests.

## Authors' contributions

HZ designed research; QJ and PZ carried out the molecular genetic studies; QJ and PZ analyzed data; QJ wrote the paper. All authors read and approved the final manuscript.
